# An Engineered Distant Homolog of *Pseudomonas syringae* TTSS Effector From *Physcomitrella patens* Can Act as a Bacterial Virulence Factor

**DOI:** 10.3389/fmicb.2018.01060

**Published:** 2018-06-20

**Authors:** Marcin Piechocki, Fabian Giska, Grzegorz Koczyk, Marcin Grynberg, Magdalena Krzymowska

**Affiliations:** ^1^Institute of Biochemistry and Biophysics (PAS), Laboratory of Plant Pathogenesis, Warsaw, Poland; ^2^Institute of Plant Genetics (PAS), Department of Biometry and Bioinformatics, Poznań, Poland; ^3^Institute of Biochemistry and Biophysics (PAS), Department of Biophysics, Warsaw, Poland

**Keywords:** HopQ1, *Pseudomonas syringae*, evolution, *Physcomitrella patens*, horizontal gene transfer, type three effector

## Abstract

*Pseudomonas syringae* pv. *phaseolicola* is the causative agent of halo blight in common bean (*Phaseolus vulgaris*). Similar to other pathogenic gram-negative bacteria, it secrets a set of type III effectors into host cells to subvert defense mechanisms. HopQ1 (for Hrp outer protein Q) is one of these type III effectors contributing to virulence of bacteria. Upon delivery into a plant cell, HopQ1 undergoes phosphorylation, binds host 14-3-3 proteins and suppresses defense-related signaling. Some plants however, evolved systems to recognize HopQ1 and respond to its presence and thus to prevent infection. HopQ1 shows homology to Nucleoside Hydrolases (NHs), but it contains a modified calcium binding motif not found in the canonical enzymes. CLuster ANalysis of Sequences (CLANS) revealed that HopQ1 and alike proteins make a distinct group of putative NHs located distantly from the classical enzymes. The HopQ1 – like protein (HLP) group comprises sequences from plant pathogenic bacteria, fungi, and lower plants. Our data suggest that the evolution of HopQ1 homologs in bacteria, fungi, and algae was independent. The location of moss HopQ1 homologs inside the fungal clade indicates a possibility of horizontal gene transfer (HGT) between those taxa. We identified a HLP in the moss *Physcomitrella patens*. Our experiments show that this protein (referred to as PpHLP) extended by a TTSS signal of HopQ1 promoted *P. syringae* growth in bean and was recognized by *Nicotiana benthamiana* immune system. Thus, despite the low sequence similarity to HopQ1 the engineered PpHLP acted as a bacterial virulence factor and displayed similar to HopQ1 virulence properties.

## Introduction

Plant pathogens have evolved several mechanisms to overcome host defense responses. One of these mechanisms relies upon the delivery of proteinaceous effectors into plant cells. Effectors are evolutionary shaped to target key elements of host immune signaling pathways. Plants in turn, evolved systems that sense some of these effectors and their perception initiates downstream signaling. It implicates that one effector may promote bacterial growth in one plant species whereas in other its presence may betray bacterial invasion to plant surveillance system. In most cases, however, plant receptors do not directly interact with their cognate effectors but rather sense specific modifications of the virulence targets introduced by the effectors (Cui et al., 2015).

*Pseudomonas syringae* is a widespread bacterium that can infect almost 200 plant species including important vegetable crops. Like many other gram-negative pathogenic bacteria, *P. syringae* injects type III effectors into host cells. The effectors contribute to disease development *via* manipulation of host defense system and physiology for the pathogen’s benefit. As a whole species, *P. syringae* produces nearly 100 different effector families (Lindeberg et al., 2012; Büttner, 2016). Their number in particular strains is variable, with a minimal known effector repertoire restricted to nine proteins (Baltrus et al., 2011). The effectors shared between most strains are called core effectors and were possibly acquired before pathovars diversification (Rohmer et al., 2004). In addition to this process of vertical inheritance, the effector repertoires are shaped by horizontal gene transfer (Rohmer et al., 2004). Acquisition of a new effector may affect bacterial virulence properties in several ways. It may expand the range of host virulence targets, contribute to host shift or mask the function of another effector to avoid host recognition.

HopQ1 (*Hrp*/*hrc*-dependent outer protein Q) is an effector recently acquired by *P. syringae* (Rohmer et al., 2004). The central region of HopQ1 shows overall homology to nucleoside hydrolases (nuc_hydro2). In contrast to the *bona fide* NH involved in purine/pyrimidine salvage pathways, the putative calcium-binding site within the predicted catalytic center of HopQ1 (DXXXDXDD) differs from the consensus sequence (DXDXXXDD). suggesting that the HopQ1 catalytic center may have evolved to process other substrates. Consistently, the purified recombinant HopQ1 neither cleaved nor bound standard substrates (Giska et al., 2013; Li et al., 2013) but it was shown by Hann et al. (2014) to hydrolyze *in vitro* the cytokinin precursor [iP-riboside 5′-monophosphate (iPRMP)].

Here we show that HopQ1 belongs to an old, widely spread protein family. To get insight into its evolution, we employed various approaches. Classical methods to infer horizontal gene transfer events are based on sequence composition or evolutionary history (Ravenhall et al., 2015). Due to high sequence divergence of HLPs (HopQ1 – like proteins) the composition based methods failed to identify HGT events. Topology of the phylogenetic tree did not allow us to exclude the hypothesis that HLPs belonging to various taxonomic groups evolved independently except for moss and fungal proteins that display strong grouping (60%). Using a HopQ1-like sequence from an unrelated organism, we performed experimental horizontal gene transfer. HLP from *P. patens*, engineered to mimic a bacterial TTSS effector displayed strong virulence properties in *Phaseolus vulgaris* but unexpectedly it triggered also defense response in *Nicotiana benthamiana* thereby determining *P. syringae* host range in a similar way to HopQ1. This suggests a functional equivalence of these two proteins.

## Materials and Methods

### Exploratory Analysis of HLP Subset Within the Nucleoside Hydrolase Superfamily

Initially, sequences homologous to HopQ1 were found using *jackhmmer* online server^1^ (Finn et al., 2011). The search was performed against non-redundant (nr) database (up-to-date in January, 2015). Two iterations were performed, as additional rounds caused the inferred profile to lose similarity to the query protein (lowering the score of the corresponding hit). In order to reduce redundancy, all of the obtained hits were clustered by the CD-HIT online tool^2^ (Huang et al., 2010) with default options active (sequence identity cut-off at 90%). The clustered sequences were checked manually and sequences truncated or lacking HopQ1-like or classical aspartic acid motif were removed.

The sequences from the database obtained previously were further supplemented by more HLPs from genome sequencing projects and all of the subjected to CLANS analysis^3^ (Frickey and Lupas, 2004) at the default options active. In particular, several fungal genome projects were searched via JGI/MycoCosm BLAST (E-value 1e-20, followed by a manual inspection) interface and draft moss genomes available at the time investigated for presence of *P. patens*-like HLP homologs (February, 2015). After that, main protein groups (clans) were manually annotated with taxonomic data available at NCBI/Taxonomy database.

### Phylogenetic Analyses of HLPs

The amino acid sequences of HLPs and RihA (used as a rooting sequence) were aligned using MAFFT-L-INS-i (Katoh and Toh, 2008), manually adjusted to contain nucleoside hydrolase domain only (based on NCBI/CDD domain boundaries), then MAFFT-L-INS-i tool was performed again. After that, the alignment was evaluated in T-COFFEE-TCS (Chang et al., 2014). The conserved core was kept for further analysis (all residues with TCS score equal or greater than 6).

The previously aligned sequences were put into Bayesian analysis in PhyloBayes version 1.5 (Lartillot et al., 2009) using implemented CAT and CAT-GTR models. Three independent chains were run for 40000 iterations and topological convergence criteria were assessed with PhyloBayes’ bpcomp and tracecomp tools. Chains were sampled at every 5th iteration and first 5000 topologies were discarded as burnin. The best converged pair of chains was selected for consensus topology calculations and based on the support values and convergence criteria CAT-GTR result was chosen. Converged values of maximum and mean difference in supports were respectively: 0.12 and 0.01, with discrepancies for all trace parameters below 0.1 and corresponding effective sample sizes all above 1000. The tree was rooted with classical nucleoside hydrolase RihA from *Escherichia coli* (GenBank Library Accession No. CQR80250.1) as an outgroup and the visualization and annotation of the resulting tree was performed using MEGA7 (Kumar et al., 2016).

### *P. syringae* Strains and Inoculation

*Nicotiana benthamiana*, tobacco (*Nicotiana tabacum* cv. ‘Xanthi-nc’), and common bean (*Phaseolus vulgaris* ‘Red Mexican’) plants were grown in soil under controlled environmental conditions (21°C, 16 h of light, 8 h of dark), as described previously (Talarczyk et al., 2002). A sequence encoding PpHLP (GenBank Library Accession No. XP_001774397.1) was optimized for bacterial systems using online server *OPTIMIZER*^4^ (Puigbò et al., 2007) with ‘*guided random*’ option applied. Then, convenient restriction sites (absent in the modified sequence) were designed. The modified sequence with the restriction sites was synthesized in GENEART^5^. A sequence encoding the TTSS signal of HopQ1 (Guttman et al., 2002) was added upstream to *PpHLP, rihA, or mCherry sequences* using primers containing appropriate restriction sites (Supplementary Table S1). The PCR product obtained and the restriction fragments containing *PpHLP, rihA, or mCherry* were triple ligated into a broad-host-range pBBR1MCSXpTAC vector (Giska et al., 2013). The constructs were electroporated into appropriate *P. syringae* strains.

*Pto*DC3000Δ28, a mutant strain of *P. syringae* pv. *tomato* DC3000 with 28 effector genes deleted (Kvitko et al., 2009) was used for virulence assays. The bacteria expressing HopQ1 or PpHLP were mixed at equal colony forming units (cfu) prior to inoculation (10^5^ cfu mL^-1^). Bacterial suspensions were infiltrated into leaves of 2-week-old bean ‘Red Mexican’ plants using a needleless hypodermic syringe. At selected time points, two 1-cm diameter leaf disks were cut from infiltrated zones in each plant. Disks were superficially sterilized with 70% ethanol for 1 min, rinsed with sterile water for 1 min, and then ground in 300 mL of 10 mM MgCl_2_. Serial dilutions were plated onto LB agar plates. The bacteria were grown at 28°C and after 2 and 6 days replicated onto plates containing either kanamycin or gentamicin, which enabled strain differentiation and cfu counting. The competitive index (CI) was calculated as described previously (Macho et al., 2007). CI was defined as the ratio of the colonies corresponding to the strain carrying PpHLP to the strain expressing HopQ1 within the output samples, divided by the corresponding ratios in the input inocula. The results were compared statistically by Student’s *t*-test, and differences were considered significant at *P* < 0.05.

For avirulence tests, the plasmid encoding PpHLP was transformed into *P. syringae* pv. *syringae* B728a strain. The bacterial cultures were adjusted to 10^6^ cfu mL^-1^ in MilliQ water and supplemented with Silwet L-77 (0.02%). Five-week-old *N. benthamiana* plants were dip inoculated by inverting whole plants into bacterial suspensions and gently agitating for 30 s. Following inoculation, plants were placed immediately under a plastic dome to maintain high humidity levels for 24 h. Development of symptoms was assessed within 7 days.

## Results

### HLPs Are Widely Distributed and Make a Distinct Group Among Nucleoside Hydrolases

It was inferred from the previous studies that HopQ1 has been recently acquired by *P. syringae* (Rohmer et al., 2004). In order to gain insight into its evolutionary history we analyzed the family of HopQ1 – like proteins (HLP). *Jackhmmer* analysis revealed over 3,600 sequences similar to HopQ1 containing non-canonical calcium binding site. Interestingly HLPs were not confined to plant pathogenic bacteria (e.g., *P. syringae, Ralstonia* spp., *Xanthomonas* spp., *Acidovorax* spp.) and fungi (e.g., *Botryotinia fuckeliana, Marssonina brunnea, Sclerotinia sclerotiorum*), but they were also found in algae (including *Aureococcus anophagefferens, Thalassiosira* sp., *Emiliania huxleyi*) and amongst several mosses (*Physcomitrella patens, Pohlia nutans, Ceratodon purpureus*).

**FIGURE 1:**
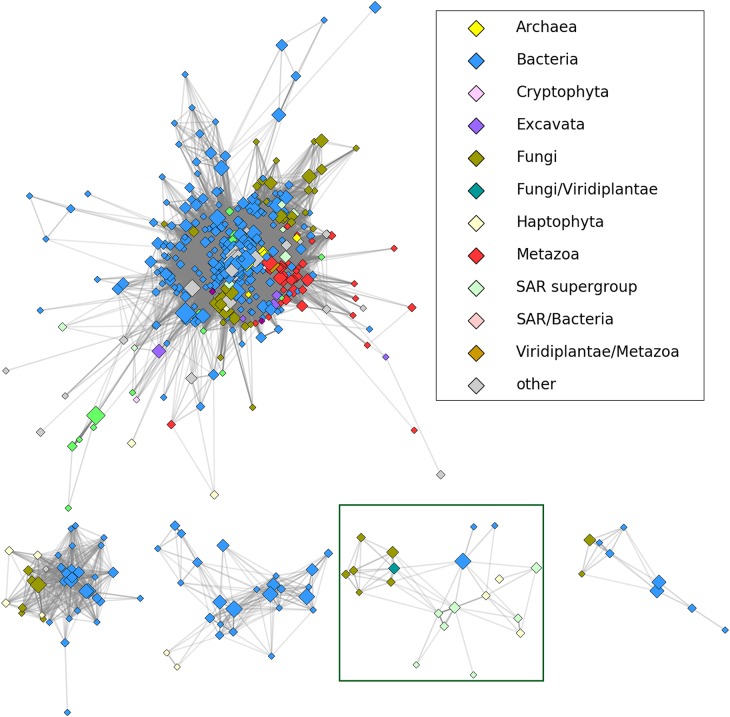
The results of CLANS clustering of nucleoside hydrolase homologs at 1E-30 E-value threshold. HopQ1 amino acid sequence was used as a query to search for homologs using *jackhmmer* tool (https://www.ebi.ac.uk/Tools/hmmer/search/jackhmmer; sequence significance E-value - 0.01; hit significance E-value - 0.03). After two iterations, CD-HIT (http://weizhongli-lab.org/cdhit_suite/cgi-bin/index.cgi?cmd=cd-hit) (Huang et al., 2010) clustering at 90% sequence identity cut-off and manual reduction of the number of hits, the remained sequences were analyzed by CLANS (https:// toolkit.tuebingen.mpg.de/#/tools/clans) program from the same toolkit. The CLANS program allows to join sequences with high homology and closest evolutionary connection into groups (clans). The five largest clans are depicted including HopQ1 homologs (HLPs). A reduced representation (sequences grouped at 70% percentage identity with CD-HIT) was used to visualize the connections in CytoScape (Shannon et al., 2003). Node color corresponds to taxonomic classification, node size is proportional to the number of sequences grouped (logarithmic scale). Shading of the edges corresponds to the best BLAST E-value (stronger similarities equal darker edges). The initial visualization was obtained in CytoScape using edge-weighted, spring-based layout and the final positions were manually adjusted for clarity. The HopQ1 clan is surrounded by a green line.

To determine a position of HLPs in relation to other members of the nucleoside hydrolase superfamily we employed CLANS, a sequence similarity-based clustering method, which groups evolutionarily closest proteins together and then places them into a three-dimensional diagram (Frickey and Lupas, 2004). To this end, all the *jackhmmer* hits were clustered in CD-HIT, manually edited (Supplementary Table S2) and then subjected to CLANS. As shown in **Figure 1** all the HLPs, containing the HopQ1-like calcium binding motif, grouped as, a distinct clan within the nucleoside hydrolase superfamily, that was distant from the classical core. This suggests that HLPs form a novel family of the proteins. Consistently, modeling of a few HLPs (Supplementary Figure S1) revealed that they share a common fold with RihA, a inosine/uridine-preferring nucleoside hydrolase from *Escherichia coli*, however their predicted structures are dissimilar to the classical nucleoside hydrolases.

### HLPs Evolved Independently At Least Three Times – An Unexpected Alliance of Mosses and Fungi

**FIGURE 2:**
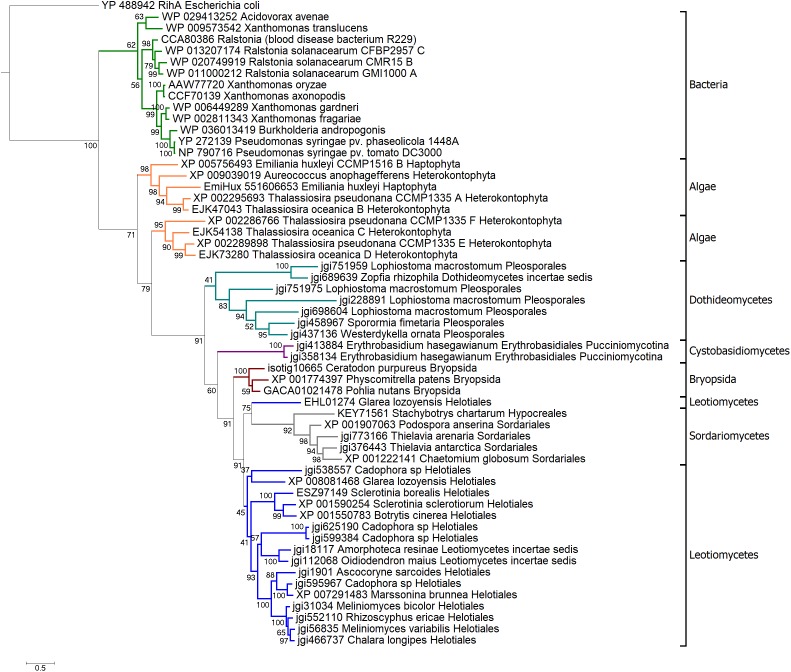
Bayesian phylogenetic tree of HLPs. The tree was rooted with classical nucleoside hydrolase RihA from *Escherichia coli*. Branches representing main taxa were highlighted: bacteria in green, algae in orange, *Dothideomycetes* in teal, *Sordariomycetes* in gray, *Cystobasidiomycetes* in purple, mosses in brown, *Leotiomycetes* in navy. Each taxon is marked with black bracket. The analysis was carried out in PhyloBayes-MPI v. 1.5 (Lartillot et al., 2009). Three chains of 40000 iterations were run in parallel, two chains of best convergence were chosen for the consensus tree construction.

To reconstruct evolutionary history of HLPs we built phylogenetic trees based on their NH domains. Amino acid sequences of HopQ1-like cluster homologs (Supplementary Table S2) were adjusted to comprise only the NH-like domain and the final alignment was performed (Supplementary Figure S2). On basis of a final, curated set of 56 sequences, a Bayesian phylogenetic tree was built. As shown in **Figure 2**, HLPs grouped in three large clades: bacterial, algal, and fungal. This suggests that in bacteria, algae, and fungi HLPs evolved largely independently. Bacterial clade consisted of the sequences coming from plant pathogens. HopQ1 formed the youngest branch of the tree. This is in line with the fact that HopQ1 acquisition by *P. syringae* occurred later than the recruitment of its homologs from other plant pathogens such as *Xanthomonas* spp. and *Ralstonia* spp. belonging to the core effectors (Rohmer et al., 2004). The sequences from heterokonts and haptophytes, including harmful brown tides causing agent *A. anophagefferens*, formed algal clade. Last clade was predominantly fungal, containing sequences from ascomycetes and basidiomycetes, including plant pathogens, saprotrophs, and mycorrhizal symbionts. Interestingly, moss clade was localized inside the fungal group. High bootstrap value supported the alliance between mosses and fungi in this context, with additional evidence pointing to nesting of the clade in between two groups of the fungal sequences (**Figure 2**). Thus the location of the moss clade indicated a possible horizontal gene transfer between those two distant groups.

### HLP From *P. patens* Promotes Faster Bacterial Growth in Common Bean Than HopQ1

**FIGURE 3:**
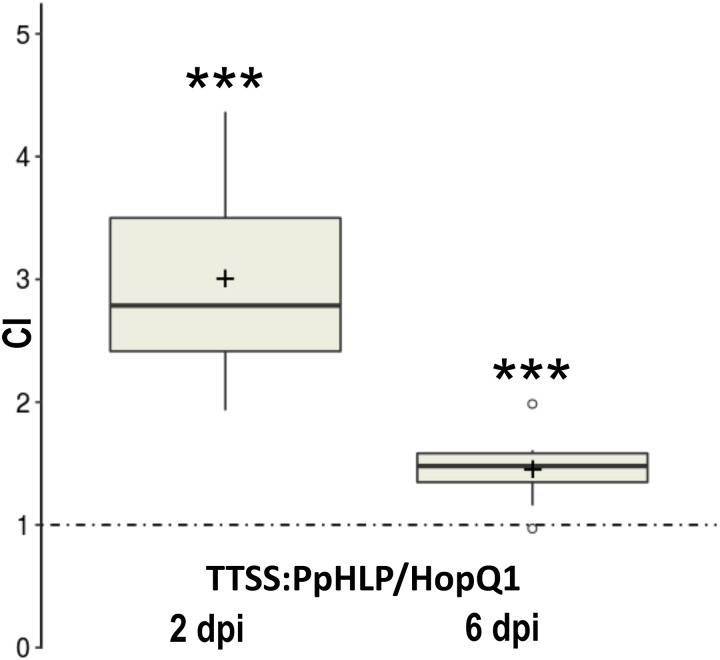
Assessment of virulence properties of TTSS-PpHLP. Bean leaves were inoculated with *Pseudomonas syringae* pv. *tomato* DC3000Δ28E (approximately 10^5^ cfu mL^-1^) strains expressing HopQ1 or TTSS-PpHLP. Immediately prior to infiltration, bacteria were mixed in a 1:1 ratio. Two and 6 days post-inoculation (dpi), two leaf disks per plant were cut out from the infiltrated zones, ground in sterile 10 mm MgCl_2_, diluted, and plated on LB medium. Bacterial strains were distinguished by a selectable marker. The CI (competitive index) was calculated as the ratio of bacteria expressing TTSS-PpHLP to bacteria expressing wild-type HopQ1 isolated from plant leaf and normalized to the input titers of the bacteria. Asterisks indicate that the index is significantly different from 1, as established using Student’s *t*-test (*P* < 0.001). Pluses represent the means. The experiment was performed three times with similar results.

We asked further whether a HLP derived from an unrelated organism may perform a similar to HopQ1 biological function. To address this question we chose a HopQ1 homolog from moss *P. patens*, that is an established model organism with a well-annotated fully sequenced genome. The sequence encoding HLP from *P. patens* (PpHLP for *P. paten*s HopQ1-like protein) was optimized for expression in bacterial systems. To enable its delivery to plant cells by *P. syringae*, a sequence encoding the N-terminal domain of HopQ1, comprising the TTSS secretion signal, was added upstream to *PpHLP* (Guttman et al., 2002). To assess virulence properties of the chimeric TTSS:PpHLP, we employed competitive index assay that compares growth of two bacterial strains in mixed infection (Macho et al., 2007). To this end, the plasmid expressing TTSS:PpHLP was introduced into *P. syringae* pv. *tomato* DC3000Δ28E, a strain deficient in 28 native effectors (Cunnac et al., 2011). Subsequently, bean leaves were infiltrated with a mixed inoculum (in a 1:1 ratio) of *P. syringae* strains expressing HopQ1 (Giska et al., 2013) and chimeric TTSS:PpHLP. At selected time points, bacteria were isolated from leaf tissue and plated onto solid media containing appropriate antibiotics. CI was calculated as the TTSS:PpHLP-to-HopQ1 ratio within an output sample normalized for bacterial load. CI value of greater than 1 indicates the strain expressing TTSS:PpHLP proliferated significantly better than the one producing HopQ1, especially at the early phase of the infection (**Figure 3**). This indicates that TTSS:PpHLP strongly promoted virulence of *P. syringae* in bean.

### TTSS:PpHLP Is Recognized by *N. benthamiana* Immune System

**FIGURE 4:**
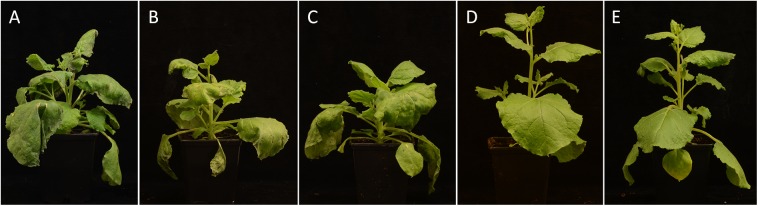
PpHLP is recognized by plant immune system. *Nicotiana benthamiana* plants were inoculated with *Psy*B728a wild-type strain **(A)** or strains carrying pBBR1-MCS2 derivatives, which express TTSS-mCherry **(B)**, TTSS-RihA **(C)**, HopQ1 **(D)**, or TTSS-PpHLP **(E)**. Disease symptoms developed only in control plants, that is plants treated with the wild-type *Psy*B728a, *Psy*B728a carrying plasmids encoding TTSS-mCherry or TTSS-RihA. The photographs were taken 7 days post-inoculation. The experiment was performed twice, with similar results.

To check whether PpHLP engineered to resemble the bacterial virulence factor is recognized by plant immune system, TTSS:PpHLP expressing plasmid was transformed into *P. syringae* pv. *syringae* B728a, which highly virulent toward many species, including *N. benthamiana* (Vinatzer et al., 2006) and does not encode HopQ1. *N. benthamiana* plants were inoculated by dipping in bacterial suspensions, and then incubated for 7 days. Non-transformed bacteria, used as a control, caused severe disease symptoms in infected plants, which eventually died (**Figure 4**). As additional controls we prepared constructs encoding mCherry or RihA preceded by the TTSS of HopQ1 and we observed similar phenotypes for the plants infected with *Psy*B728a carrying them. As previously shown, expression of HopQ1 rendered the bacteria avirulent due to HopQ1 recognition by *N. benthamiana* immune system (Giska et al., 2013). Surprisingly, inoculation with the strain expressing TTSS:PpHLP did not cause any macroscopic disease symptoms – alike HopQ1 expressing strain. This indicates, that PpHLP, despite low sequence similarity to HopQ1 (25% protein identity), triggers plant immune systems.

## Discussion

Previous bioinformatical studies showed, that proteins homologous to HopQ1 are widely spread among many species of plant pathogenic bacteria (Rohmer et al., 2004). In some genera, including *Xanthomonas* and *Ralstonia*, HopQ1 homologs belong to the core sets of effectors and are chromosomally encoded (Rohmer et al., 2004). In contrast, HopQ1 is present only in some *P. syringae* strains and it can be encoded either on the chromosome (e.g., *P. syringae* pv. *tomato*) or on a plasmid (e.g., *P. syringae* pv. *phaseolicola*). Moreover, GC content, codon usage and presence of remnants of mobile elements in the neighborhood of *hopQ1* sequence indicate a recent acquisition of this effector by *P. syringae* (Rohmer et al., 2004). This is consistent with HopQ1’s position as the youngest branch of the bacterial clade in Bayesian tree of HLPs, supported by high bootstrap values (99) (**Figure 2**).

Our analyses showed that proteins homologous to HopQ1 can be found not only in plant pathogenic bacteria, but they also occur in fungi and lower plants such as algae and some moss species. Bayesian tree of HopQ1 homologs revealed at least three large clades consisting of bacterial, algal, and fungal proteins respectively. These groups are supported by high bootstrap values (>60), which suggest that they evolved independently. What is interesting, the moss subclade is localized inside a bigger one, which contains fungal sequences. It is also well-supported by high bootstrap value (91). This may indicate a possible horizontal gene transfer between those two taxa.

Fungi interact with mosses by different means, and fungal-to-plants gene transfers have been already reported (Richards et al., 2009). Various fungal species may infect mosses causing severe disease symptoms (Akita et al., 2011). Some, like *Oidiodendron maius* – a mycorrhizal symbiont of ericaceous plants – can live as endophytes and/or saprobes on *Sphagnum* peat moss (Thormann et al., 2002; Davey and Currah, 2006). Arbuscular mycorrhizal fungi also associate with mosses (Zhang and Guo, 2007).

When did some moss species obtain fungal (or fungal-like) HopQ1 homolog? The available evidence suggests that it might have occurred in early phase of land colonization by plants, before radiation of ascomycetes, at least 400 mya (millions of years ago). More than that, the presence of ‘HopQ1’ – encoding sequence in *Erythrobasidium hasegawianum* genome suggests that HGTs might have happened even earlier, prior to the diversification of ascomycetes and basidiomycetes, which occurred ca. 600 mya.

We have conducted an experimental HGT by introducing a sequence encoding HopQ1 homolog from moss *P. patens* (PpHLP) into *P. syringae*. This resulted in a significantly enhanced bacterial growth rate in bean, compared to *hopQ1* carrying bacteria (**Figure 3**). Such scenario is usual for new, highly virulent pathogen species or strains, which did not co-evolve with their actual host. Unexpectedly, despite the low sequence similarity to HopQ1 (25% identity), expression of TTSS:PpHLP induced defense response in *N. benthamiana* plants (**Figure 4**). This was possibly not due to the fragment of HopQ1 containing the secretion signal (TTSS) that had been added N-terminally to PpHLP, since it was previously shown no to be recognized in *Nicotiana* spp. (Li et al., 2013). Moreover, previous studies indicated that no particular motif in HopQ1 sequence triggered defense response in *N. tabacum* leaves and it was inferred that the whole protein is required to induce immunity (Li et al., 2013; Hann et al., 2014). Together with the fact, that receptor Roq1 (Recognition of XopQ 1) of *N. benthamiana* mediates recognition of both HopQ1 and its close homolog from *Xanthomonas* spp. XopQ (Schultink et al., 2017), this would suggest the recognition of HLPs is indirect. In summary, introduction of the new virulence factor into *P. syringae* was advantageous to bacteria infecting the susceptible host, however the plant strategy to guard virulence targets turned to be successful in recognition of an unrelated factor by the resistant host.

The available phylogenetic evidence points to a monophyletic descent of the major clades (bacterial, two microalgal clades, and fungal/moss clade). Likewise an initial, exploratory analysis of base/codon compositions (principal components analysis in the sequence space) did not provide decisive argument regarding HopQ1 homolog origins in the aforementioned groups.

Concerning the HopQ1 homologs from moss, there is a clear evidence of grouping of the fungal and moss sequences (**Figure 2**). However, the nesting order does not provide sufficient support for horizontal transfer (the moss clade nested within the fungal sequences, with support of 60% in the final Bayesian consensus) rather than duplication (the moss clade located at the crown of the fungal clade). Following the principle of maximum parsimony we are thus forced to conclude that while both modeling and functional evidence from the “artificial” horizontal transfer corroborate a possibility of ancient transfers, the available genomic evidence was insufficient for a definite conclusion. In summary, for major clades from bacterial and eukaryotic microorganisms that are parallel, vertical descents from a common ancestor sequence remain a possibility.

## Author Contributions

FG, MP, GK, and MK conceived and designed the experiments. MP, FG, and GK performed the experiments. FG, MP, GK, MG, and MK analyzed the data. MP and MK wrote the paper.

## Conflict of Interest Statement

The authors declare that the research was conducted in the absence of any commercial or financial relationships that could be construed as a potential conflict of interest.

## Footnotes

1 https://www.ebi.ac.uk/Tools/hmmer/search/jackhmmer

2 http://weizhongli-lab.org/cdhit_suite/cgi-bin/index.cgi?cmd=cd-hit

3 https://toolkit.tuebingen.mpg.de/#/tools/clans

4 http://genomes.urv.es/OPTIMIZER/

5 https://www.thermofisher.com/pl/en/home/life-science/cloning/gene-synthesis/geneart-gene-synthesis.html
