# Supplementary material for: An Engineered Distant Homolog of Pseudomonas syringae TTSS Effector From Physcomitrella patens Can Act as a Bacterial Virulence Factor
Source: Front Microbiol. 2018 Jun 20;9:1060. doi: 10.3389/fmicb.2018.01060 (PMC6019455; doi:10.3389/fmicb.2018.01060)
Supplement: Supplementary file 1 [file Data_Sheet_1.PDF]

Supplementary Material

An engineered distant homolog of *Pseudomonas syringae* TTSS effector from *Physcomitrella patens* can act as a bacterial virulence factor

Marcin Piechocki[1], Fabian Giska[1], Grzegorz Koczyk[2], Marcin Grynberg[3], Magdalena Krzymowska[1*]

*** Correspondence:** Magdalena Krzymowska: krzyma@ibb.waw.pl

Supplementary Table 1. Primers used in this work.

**physco-topo-F**<br>**physco-topo-R**	CACCATGGCCGCGTCGAGTACCAGG<br>CAAGTTACTATAACGTAAAGCGTCAAG	Cloning of cDNA of PpHLP to pENTR D-TOPO vector
**W-phys-F**<br>**W-phys-R**	GAGGGTCCATCATTGGATCGCAGACGATGAG<br>ACTACCTTCCCTGTCGACTTCAACTC	Sequencing of PpHLP
**physco-bact-seq**<br>**W-phys-bact-F**<br>**W-phys-bact-R**	CCTCCGAGGTATTTGTAG<br>ACCGGACGAAGAAGAAGAACTGG<br>GGGTGACCAGACGCTTCCAGTTC	Sequencing of the recoded PpHLP
**phys-stop-F**<br>**phys-stop-R**	CAGCAACCTGTAGTGACTCGAGGGTACC<br>TAGCGCAGGGCATCCAGCAGAGAACC	Adding STOP codon to sequence of recoded PpHLP
**TTSS-F**<br>**TTSS-R**<br>**TTSS-R-Nco**	GGATCCATGCATCGTCCTATCAC<br>CATATGAGTGAAAAACCACGTATCCTTAG<br>CCATGGAGTGAAAAACCACGTATCCTTAG	Adding secretion signal to pBBR1MCSXpTAC vector
**physco-ef-topo-F**<br>**physco-ef-topo-R**	CACCATGGATCGTCCTATCACCGCAGGCCATAC<br>CAGGTTGCTGTAGCGCAGGGCATCC	Cloning a coding sequence of chimeric protein (TTSS-recoded PpHLP) to pENTR D-TOPO vector

Supplementary Figure 1

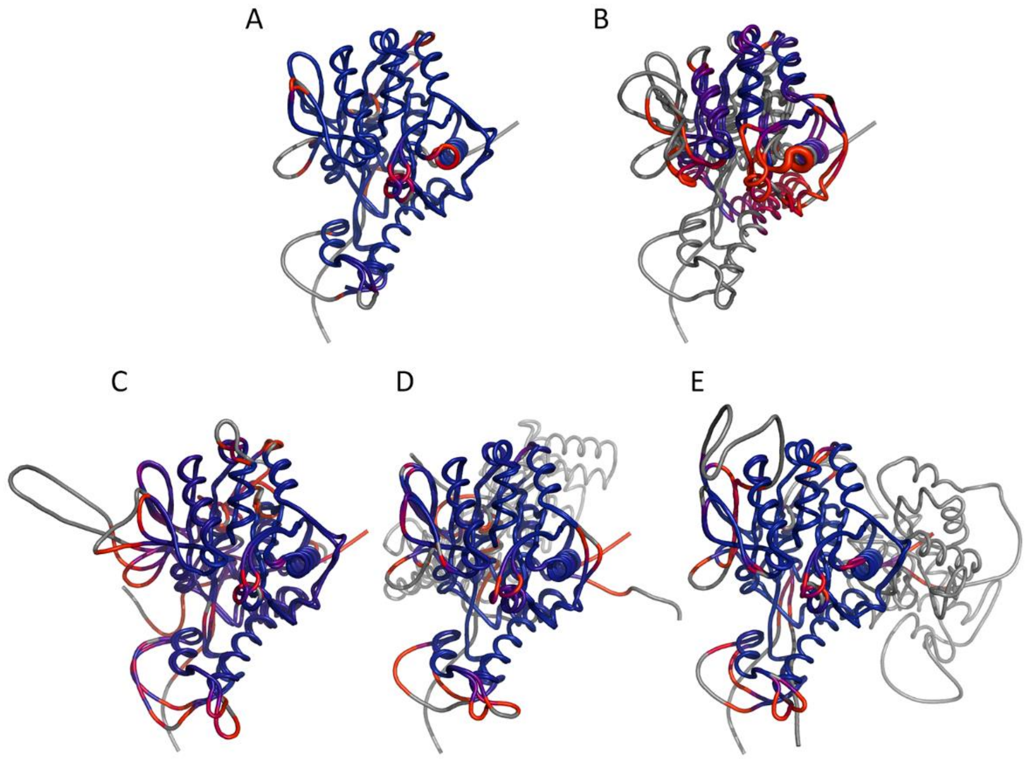

Visualization of the structural alignments between PpHLP (*P. patens* HopQ1 homolog; XP_001774397) and other clan members. The models were derived from RAPTORX modeling with the default settings (Kallberg et al., 2012). Alignment was carried out using CEALIGN algorithm (Jia et al., 2004) as implemented in PyMol 1.8.0 and coloring was done based on the obtained RMSD distances (spectrum-based coloring, blue denotes the best aligned regions, red – the worst aligned regions with RMSD equal or more than 5A, the unaligned regions are colored in gray). Note that microalgae (*Aureococcus anophagefferens* XP_009039019, *Thalassiosira oceanica* EJK54138.1) homologs are double domain structures and one of the domains is depicted as unaligned for this visualization.A - superposition of *Xanthomonas oryzae* XopQ (PDB:4P5F) with the predicted protein XP_001774397.1 from *Physcomitrella patens* model; B - superposition of rihA (PDB:1YBE) model with the predicted protein XP_001774397.1 from *Physcomitrella patens* model; C – superposition of predicted protein XP_001550783 from *Botrytis cinerea* with the predicted protein XP_001774397.1 from *Physcomitrella patens* model; D – superposition of hypothetical protein THAOC_26301(EJK54138.1) with the predicted protein XP_001774397.1 from *Physcomitrella patens* model; E – superposition of hypothetical protein AURANDRAFT_65642 (XP_009039019) with the predicted protein XP_001774397.1 from *Physcomitrella patens* model

Supplementary Figure 2

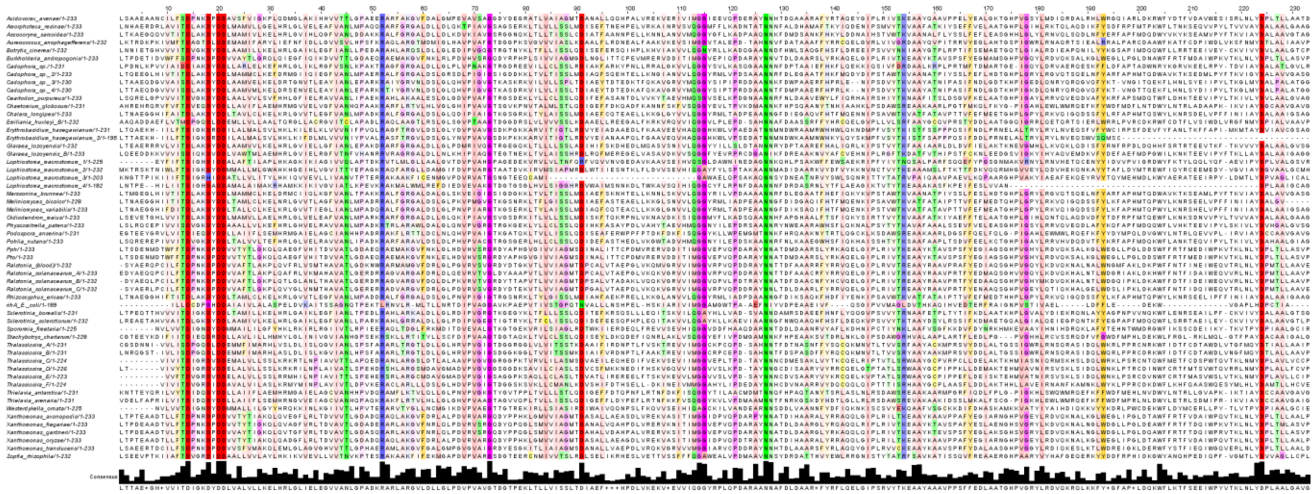

Alignment of 56 sequences belonging to HopQ1-like proteins. 56 sequences and 233 positions were aligned using MAFFT-L-INS-i (Katoh and Toh, 2008) and evaluated in T-COFFEE-TCS (Chang et al., 2014). Visualization was performed in Jalview 2.10.x (Waterhouse et al., 2009). Amino acid color scheme chosen as Zappo (I, L, V, A, M (aliphatic/hydrophobic) – pink; F, W, Y (aromatic) – orange; K, R, H (positively charged) – red; D, E (negatively charged) – green; S, T, Q (hydrophilic) – mid blue; P, G (conformationally special) – magenta; C – yellow) with default value of conservation threshold option checked.

Chang, J.M., Di Tommaso, P., and Notredame, C. (2014). TCS: a new multiple sequence alignment reliability measure to estimate alignment accuracy and improve phylogenetic tree reconstruction. *Mol Biol Evol* 31(6)**,** 1625-1637. doi: 10.1093/molbev/msu117.

Jia, Y., Dewey, T.G., Shindyalov, I.N., and Bourne, P.E. (2004). A new scoring function and associated statistical significance for structure alignment by CE. *J Comput Biol* 11(5)**,** 787-799. doi: 10.1089/cmb.2004.11.787.

Kallberg, M., Wang, H., Wang, S., Peng, J., Wang, Z., Lu, H., et al. (2012). Template-based protein structure modeling using the RaptorX web server. *Nat Protoc* 7(8)**,** 1511-1522. doi: 10.1038/nprot.2012.085.

Katoh, K., and Toh, H. (2008). Recent developments in the MAFFT multiple sequence alignment program. *Brief Bioinform* 9(4)**,** 286-298. doi: 10.1093/bib/bbn013.

Waterhouse, A.M., Procter, J.B., Martin, D.M., Clamp, M., and Barton, G.J. (2009). Jalview Version 2--a multiple sequence alignment editor and analysis workbench. *Bioinformatics* 25(9)**,** 1189-1191. doi: 10.1093/bioinformatics/btp033.
